# Why does a natural myringostapediopexy cause minimal hearing loss?

**DOI:** 10.1002/ccr3.4946

**Published:** 2021-10-18

**Authors:** Dorji Penjor

**Affiliations:** ^1^ Department of Otorhinolaryngology Jigme Dorji Wangchuck National Referral Hospital Thimphu Bhutan; ^2^ Faculty of Postgraduate Medicine Khesar Gyalpo University of Medical Sciences of Bhutan Thimphu Bhutan

**Keywords:** automastoidectomy, hearing impairment, natural myringostapediopexy, tympanic membrance retraction

## Abstract

Natural myringostapediopexy is a result of tympanic membrane retraction with automastoidectomy that causes the tympanic membrane to plaster onto the stapes directly. It causes minimal conductive hearing impairment.

## Graphical abstract

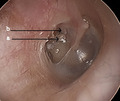

## 1 DESCRIPTION

Natural myringostapediopexy is an uncommon occurrence. There is retraction of the tympanic membrane with automastoidectomy, and the tympanic membrane sits directly on the stapes. Conductive hearing impairment is mild. The intent of this clinical image is to illustrate the otoscopic findings of a natural myringostapediopexy.

There are different types of surgical procedures called tympanoplasty to improve hearing impairment caused by the diseased tympanic membrane or the ossicles. As per Wullstein classification, type III tympanoplasty is the repair of the tympanic membrane and its placement on the stapes head when there is a defect in the malleus and incus. Myringostapediopexy is a type III tympanoplasty that can also occur naturally. Why does natural myringostapediopexy cause minimal hearing loss?

**FIGURE 1:**
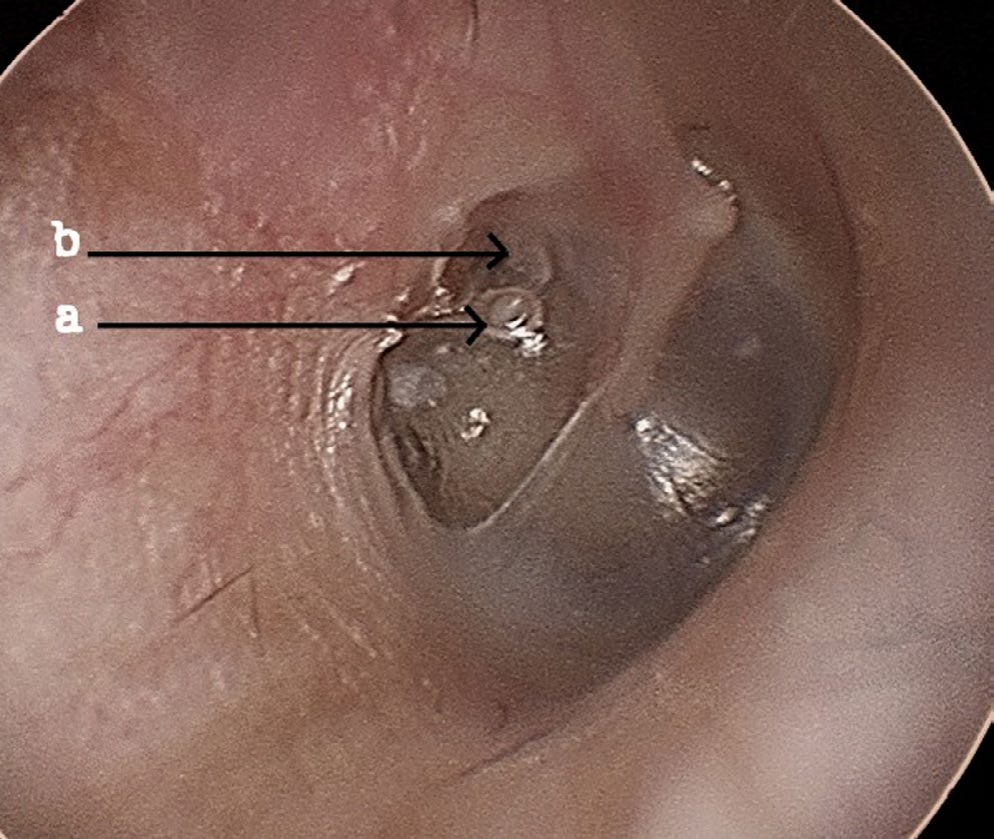
Natural myringostapediopexy

A 44‐year‐old lady complained of a ringing sound in the right ear on and off for the last 7 years. She also complained of blocked sensation in the same ear. She never had any ear surgery. Otoscopic examination of the right ear showed retraction of the posterosuperior aspect of pars tensa (Figure 1) and adherent to the stapes head (arrow a) that is dislocated from the long process of incus (arrow b).

Rinne test was positive bilaterally, and weber lateralized to the right. This is an uncommon case of natural myringostapediopexy. In a normal ear, sound travels from the tympanic membrane via the ossicular chain to the cochlea. A defect in its conductive pathway causes hearing impairment. In myringostapediopexy, the sound is conducted directly from the tympanic membrane to the stapes head. Natural myringostapediopexy is an infrequent abnormality and may function as a type III tympanoplasty with most cases having minimum conductive hearing loss.^1^ A similar range of hearing level has been found to be associated with both naturally formed and surgically fashioned myringostapediopexy with most cases having an air‐borne gap of 20 dB or less.^2^

## CONFLICT OF INTEREST

None declared.

## AUTHOR CONTRIBUTIONS

DP: concept, writing up, photography and final approval.

## ETHICAL APPROVAL

Our institution does not require ethical approval for the publication of clinical image, and the consent was obtained from the patient.

## CONSENT

Informed written consent was obtained from the patient.

## Data Availability

Data sharing is not applicable to this article as no datasets were generated or analyzed during the current study.
